# Treatment of COVID-19 Patients Post-Bariatric Surgery: Issues for Consideration

**DOI:** 10.3390/jcm9092827

**Published:** 2020-08-31

**Authors:** Carmil Azran, Daniel Porat, Arik Dahan, Dror Dicker

**Affiliations:** 1 Clinical Pharmacy, Herzliya Medical Center, Herzliya 46140, Israel; CarmilA@hmc.co.il; 2 Department of Clinical Pharmacology, School of Pharmacy, Faculty of Health Sciences, Ben-Gurion University of the Negev, Beer Sheva 8410501, Israel; poratdan@post.bgu.ac.il; 3 Department of Internal Medicine D and Obesity Clinic, Hasharon Hospital-Rabin Medical Center, 7 Keren Kayemet St., Petah-Tikva 49100, Israel; drord@clalit.org.il

**Keywords:** COVID-19, bariatric surgery, pharmacotherapy, drug absorption

## Abstract

As COVID-19 has been expanding rapidly around the world, the types of patients and their backgrounds vary. The substantially altered GI anatomy/physiology after bariatric surgery presents new challenges to the field of oral drug therapy. In this report we highlight issues for consideration when treating COVID-19 patients who previously underwent bariatric surgery and provide practical tools to allow optimal care of these patients. Post-bariatric absorption/pharmacokinetic changes may warrant dose adjustment, as well as the use of liquid oral dosage forms or parenteral routes of administration, if available. Realizing the potentially altered pharmacokinetics of various drugs after bariatric surgery is essential for providing optimal pharmacological therapy and overall patient care.

## 1. Introduction

As coronavirus disease 2019 (COVID-19) has been expanding rapidly around the world with significant morbidity and mortality, the types of patients and their backgrounds vary. Currently, with a second wave already arrived in many countries, there are several treatments that are being investigated and used on patients diagnosed with COVID-19 in hopes of reducing the duration of viral shedding and viral excretion, and reducing the severity of the disease and mortality. The pipeline for COVID-19 novel treatments is focused mainly on antiviral drugs and vaccines.

Aside from the advent of COVID-19, another wide-spread epidemic of the western world is obesity and the mainstay of treatment is currently bariatric surgery. The different bariatric procedures include gastric banding, Roux-en-Y Gastric Bypass (RYGB), sleeve gastrectomy, and more recently one-anastomosis gastric bypass. The modified gastrointestinal (GI) anatomy/physiology of bariatric patients may lead to significant changes in oral drug bioavailability, and pharmacokinetic (PK) alterations of many drugs post-surgery have been reported in the literature [1,2]. Among these drugs, the anti-viral family is particularly sensitive to bariatric-related pharmacokinetic alterations [3,4]. This phenomenon may be explained by the common physicochemical features shared by many of these agents. 

To note, while decreased or unchanged drug blood levels may be expected after bariatric surgery due to absorption issues, increased concentrations have also been reported in the literature [5,6]. These varied results largely depend on the drug in question, as each and every drug may or may not be influenced by one or more biopharmaceutical/pharmacokinetic mechanisms potentially altered after the surgery [7,8,9,10]. 

Since obesity is one of the main risk factors for severe COVID-19 disease, and as we find ourselves treating more and more COVID-19 patients, among these we find post-bariatric surgery patients, and we are faced with the challenge of treating them with the current treatments that are very limited on evidence and yet are what is currently available on hand. In this communication we wish to highlight issues for consideration and raise the awareness of medical staff when treating COVID-19 patients who previously underwent bariatric surgery.

## 2. Current Therapeutic Options for COVID-19

Among the orally administered treatments used in COVID-19 patients, hydroxychloroquine and chloroquine have been found to be efficient on SARS-CoV-2 patients. There is controversy in their use due to potential toxicity and questionable efficacy, however, due to the limited range of drugs on hand they are still used in many centers [11,12]. A small study, by Gautret et al, treated PCR-confirmed COVID-19 cases with hydroxychloroquine 200 mg three times daily for ten days and azithromycin 500 mg on day 1 followed by 250 mg per day for the next four days [13]. Corticosteroids are currently being used in the treatment of many COVID-19 pneumonia patients. These drugs modulate the inflammatory response associated with the disease. Although controversial, there is some evidence of possible reduced ICU admission and/or death associated with corticosteroids treatment [14]. Baricitinib, a JAK inhibitor used in rheumatoid arthritis, has shown some promising preliminary results as a possible adjuvant treatment in COVID-19 moderate to severe pneumonia [15]. Oral zinc is another option worthy of further investigation, after showing treatment potential in several cases when given in high doses [16]. It may also be used as an adjuvant or as prophylaxis. Another treatment with possible efficacy in treatment of COVID-19 is remdesivir, which is administered intravenously. Additional, less common treatments with possible efficacy that have been suggested for COVID-19 are lopinavir/ritonavir, ribavirin, Xuebijing, umifenovir, ibrutinib, interleukin-6 inhibitors, interferon, and intravenous immunoglobulin [17]. 

## 3. COVID-19 Treatment after Bariatric Surgery

When considering the effects of bariatric surgery on medications that are administered orally, there are limited data, yet we cannot disregard the fact that bariatric surgery, specifically bypass surgery, is likely to have an effect. 

As for hydroxychloroquine, a recent case report described three post-RYGB patients with autoimmune disease, taking standard hydroxychloroquine doses. All three patients had subtherapeutic hydroxychloroquine blood levels and active disease; therapeutic drug levels as well as better disease control were achieved only after increasing their doses above the recommended 400 mg/day dosing. Hence, it is suggested that hydroxychloroquine has inadequate absorption post-RYGB (and potentially other bariatric procedures), and COVID-19 bariatric patients may require hydroxychloroquine doses higher than recommended [18]. No information regarding chloroquine and bariatric surgery is currently available, but the high similarity between these two drugs suggests that this inadequate absorption may be relevant to chloroquine as well. 

Regarding azithromycin, a single-dose PK study in 14 post-gastric bypass patients showed that bioavailability was reduced by one-third in gastric bypass subjects compared with matched controls. The authors concluded that potential for treatment failure exists, recommending closer clinical monitoring and, potentially, dose adjustment of gastric bypass patients receiving azithromycin [19]. The authors explained the results by the bypass of the proximal gut, where the maximal absorption of azithromycin occurs. We believe that an additional mechanism may be involved in the altered PK. During RYGB, the stomach is dissected, leaving a very small (30 mL), and much less acidic, gastric pouch. Given the basic (alkaline; pKa = 8.5) nature of this drug, along with its low water solubility, the dissolution of the large, 500 mg, drug dose may be severely hampered after surgery, negatively affecting the absorption and bioavailability of azithromycin.

As for corticosteroids, the pharmacokinetics of dexamethasone and related steroids may be altered, especially after gastric bypass surgery. These drugs are mainly absorbed via the proximal gut, which is bypassed during the bariatric procedure. Glucocorticoid metabolism is also altered after bariatric surgery [20]. Clinical data are limited to one study, reporting two cases of uncontrolled cortisol levels during oral dexamethasone treatment after bariatric surgery, until dexamethasone was administered intravenously [21]. In addition, steroids may increase the risk of marginal ulcers post-bypass surgery and care should be taken to prevent them.

No data on baricitinib treatment after bariatric surgery are currently available. On the one hand, the basic (alkaline) nature of this molecule may impair its solubility in the higher stomach pH post-bariatric surgery [8]. On the other hand, given that the drug is administered in low, 2 mg doses, and that it is normally quickly absorbed with high bioavailability, absorption issues for this drug may not be present after bariatric surgery. Furthermore, only 10% of the drug is metabolized, so post-bariatric metabolic changes are also unlikely to alter the drug pharmacokinetics.

While limited data on post-bariatric drug exposure changes are currently available, more widespread research has been carried out on bariatric surgery and nutrient supplementation. Depending on the bariatric procedure, patients are subject to nutrient deficiencies, with deficiency of zinc being one of the most serious and prevalent [22]. Ruz et al. studied the zinc absorption among RYGB patients and showed impaired supplemental zinc absorption and zinc status at 6, 12, and 18 months following the surgery [23]. Zinc absorption is facilitated by binding to sulfur-containing amino acids and hydroxy acids [24], which may be lacking after bariatric surgery, resulting in zinc malabsorption.

Currently there is scarce information regarding lopinavir/ritonavir and bariatric surgery. A notable reduction in the serum levels of lopinavir/ritonavir was described in one case report of an HIV-infected pregnant woman with a gastric bypass [25]. On the other hand, with another drug combination that included ritonavir (darunavir/ritonavir), blood levels and pharmacological effect similar to pre-RYGB were described in a single case report [26]. This is in corroboration with a case report of normal blood levels and therapeutic effect of lopinavir/ritonavir in a gastrectomized patient (which shares some similarities to bariatric procedures) [27]. Since liquid drug product is available for lopinavir/ritonavir combination, it is advisable to use the oral solution rather than the tablets when treating COVID-19 patients after bariatric surgery.

## 4. Summary of Recommendations 

Due to these findings, we suggest that clinicians treating COVID-19 patients should consider that for maximal clinical effect of these medications, higher doses should be used to prevent treatment failure, possibly due to inadequate absorption. In addition, liquid drug products should be preferred over solid ones when possible. Intravenous medications of course have no issues of absorption, although these may not be on hand. 

In the case of hydroxychloroquine, the benefit vs. risk of toxicity and the patient medical history should be considered. With this in mind, if the mainstay of treatment per hospital protocol is 200 mg twice daily post-load, close clinical monitoring is required, with adjustment of dosage regimen/treatment, if indicated. If there is a possibility of drug level monitoring, that is recommended as well. In the case of azithromycin, increasing the dose by a third might be appropriate in bariatric patients. Regular ECG QT interval follow up is warranted. 

In order to overcome possible zinc malabsorption, non-oral routes of administration should be considered. Alternatively, splitting the dose may overcome the absorption issues, allowing higher systemic zinc exposure. 

Other medications lack data regarding post-bariatric surgery usage, and if using them is suggested, pharmacokinetic mechanisms and absorption sites should be considered. Awareness of the treatment challenges of these patients is important, and more clinical studies with a larger population and more treatments are warranted at this time. 
